# P-1933. MASTARD-COVID-19: A MAtched-cohort Study of Specific-Treatment-requiring Autoimmune Rheumatic Diseases after COVID-19 Using Japanese Health Insurance Claims Data

**DOI:** 10.1093/ofid/ofae631.2092

**Published:** 2025-01-29

**Authors:** Ryuichi M Sada, Shungo Yamamoto, Saki Sada Minoda, Ryotaro Tajima, Shogo Miyazawa, Takuji Komeda, Satoshi Kutsuna

**Affiliations:** Osaka University Graduate School of Medicine, Suita, Osaka, Japan; Osaka University, Suita city, Osaka, Japan; Department of Respiratory Medicine and Clinical Immunology, Graduate School of Medicine, Osaka University, Suita, Osaka, Japan; Shionogi & Co., Ltd., Chuo-Ku, Osaka-Shi, Osaka, Japan; Shionogi & Co.,Ltd, Osaka, Osaka, Japan; Shionogi & Co., Ltd., Chuo-Ku, Osaka-Shi, Osaka, Japan; Graduate School of Medicine/Faculty of Medicine, Osaka University, Suita, Osaka, Japan

## Abstract

**Background:**

Recent database studies indicate an increase in new cases of autoimmune inflammatory rheumatic diseases (AIIRDs) following coronavirus disease 2019 (COVID-19), but these studies have significant limitations. The main issue is the reliance on disease codes like ICD-10 for diagnoses, which does not ensure accuracy. Overcoming the limitation of previous studies, we studied whether a history of COVID-19 infection increases the risk of developing AIIRDs requiring disease-specific treatment.

**Figure 1.**
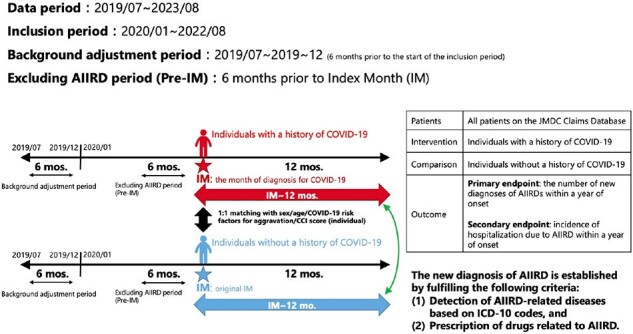
Details of the design of this matched cohort study.

**Methods:**

This matched cohort study utilized the JMDC Claims Database, sourced from health insurance union receipts from July 2019 to August 2022. Individuals with a history of COVID-19, identified by ICD-10 codes, were placed in Group 1. Group 2 consisted of individuals without a medical history of COVID-19, matched 1:1 with those in Group 1 based on sex, age, risk factors for severe outcomes, and the Charlson Comorbidity Index (Figure 1). We evaluated the incidence of AIIRDs within one year, defined by both diagnosis and the initiation of disease-specific medications (including corticosteroids, immunosuppressants, disease-modifying anti-rheumatic drugs, and biologics, etc.) that two board-certified rheumatologists reviewed. Additionally, we compared the incidences of hospitalization due to AIIRDs between the two groups.

**Table 1.**
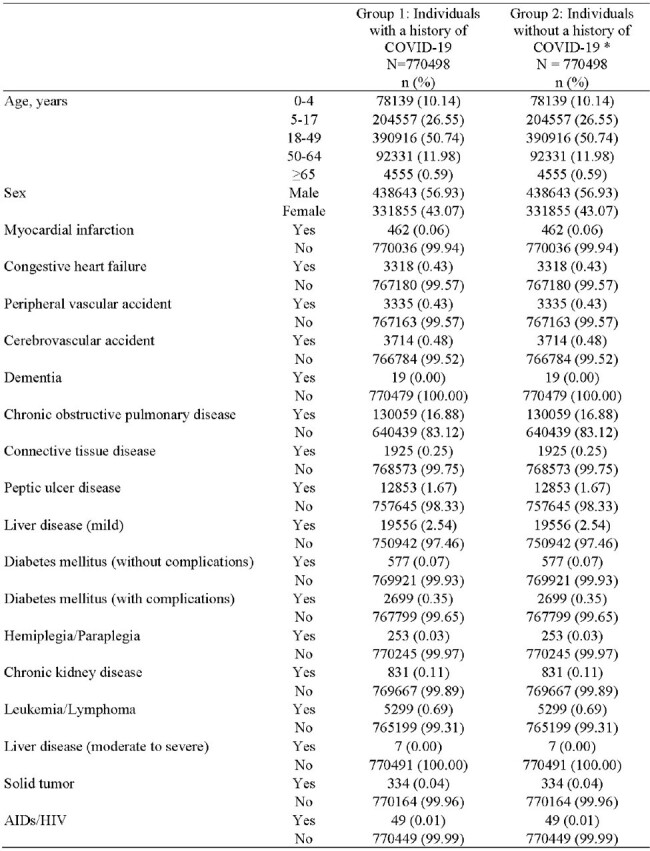
Demographics and Clinical Characteristics. *One control could have been matched to multiple patients. Abbreviations: AIDS/HIV: acquired immunodeficiency syndrome / human immunodeficiency virus.

**Results:**

Group 1 and Group 2 included 770,498 individuals in each. The patients' characteristics were presented in Table 1. Within a study period, 0.21% (1,587/766,402) in Group 1 developed AIIRDs required disease-specific treatment, and 0.11% (839/766,402) in Group 2, with a risk difference of 0.10% [95% confidence interval (C.I.): 0.085-0.110], and a risk ratio of 1.9 (95% C.I.: 1.7-2.1). The incidence of more than half of the AIIRD types was statistically significantly higher in Group 1 than in Group 2 (Figure 2). Moreover, Group 1 experienced a notably higher incidence of hospitalization due to AIIRD than Group 2, with a risk difference of 0.027 (95% C.I.: 0.023-0.030), and a risk ratio of 21 (95% C.I.: 11-40).

**Table 2.**
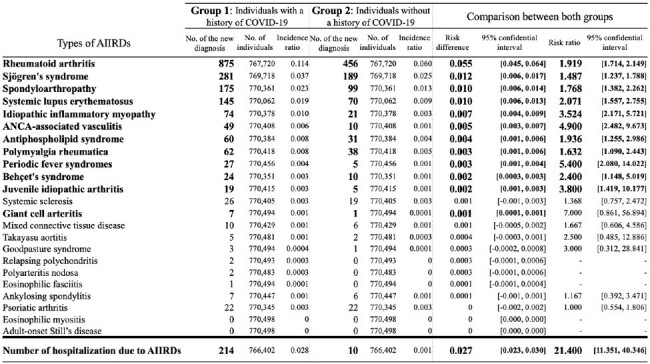
Risk differences and risk ratios of the diagnosis of treatment-requiring AIIRDs between each group. Abbreviations: AIIRDs: autoimmune inflammatory rheumatic diseases.

**Conclusion:**

Compared to the matched group of individuals without a history of COVID-19, the group with a history of COVID-19 showed a higher incidence of a variety of AIIRDs requiring specific treatments and an increased incidence of hospitalizations due to AIIRDs.

**Figure 2.**
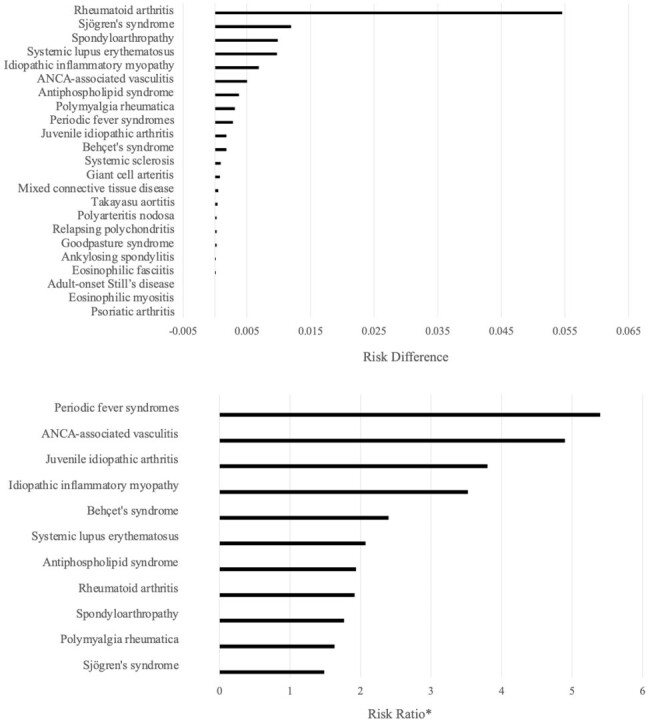
Risk differences and risk ratios of the diagnosis of AIIRDs requiring specific treatments between each group. * Risk ratios are shown only for those that can be calculated and show a significant difference.

**Disclosures:**

Ryotaro Tajima, Master of information science, Shionogi & Co., Ltd.: Employee|Shionogi & Co., Ltd.: Stocks/Bonds (Public Company) Shogo Miyazawa, MSc, Shionogi & Co.,Ltd: Stocks/Bonds (Public Company) Takuji Komeda, n/a, Shionogi & Co., Ltd.: Employee|Shionogi & Co., Ltd.: Stocks/Bonds (Public Company)

